# Pathogenesis, pathological characteristics and individualized therapy for immune-related adverse effects

**DOI:** 10.1016/j.pccm.2023.08.002

**Published:** 2023-09-13

**Authors:** Kang Miao, Li Zhang

**Affiliations:** Department of Pulmonary and Critical Care Medicine, Peking Union Medical College Hospital, Chinese Academy of Medical Sciences & Peking Union Medical College, Dongcheng District, Beijing 100005, China

**Keywords:** Immune checkpoint inhibitors, Immune-related adverse events, Pathogenesis, Pathology, Biomarker

## Abstract

Immune checkpoint inhibitors (ICIs) are a class of antitumor medications that target immune checkpoints, which induce the activation of lymphocytes. These treatments effectively prolong the survival of patients with advanced tumors, especially lung cancer. However, in addition to tumor killing effects, ICIs may also cause an imbalance between immune tolerance and immunity. Over-activated lymphocytes may cause various types of damage to multiple organs throughout the body, called immune-related adverse events. In this review, we summarize the pathogenesis, pathological characteristics, biomarkers, and therapeutic agents for immune-related adverse events.

## Introduction

To maintain homeostasis, the immune system exhibits the ability to tolerate self-antigens through central and peripheral tolerance mechanisms.^1^ The central tolerance mechanism is the negative selection for self-reactive T cell clones by the thymus. Some self-antigenic peptides are presented by antigen-presenting cells (APC) to immature T cells. The T cells that bind to these antigens with high affinity are eliminated.^2^ However, some antigenic peptides were not available in thymus. Therefore, peripheral tolerance mechanisms function as a complement. T cells are activated by the co-regulation of two signals. T-cell receptor (TCR) combined with the major histocompatibility complex (MHC) of APC mediates the generation of the first signal, while co-stimulatory molecules (e.g., CD28) bind corresponding receptors and mediate the generation of the second signal. Additionally, co-inhibitory regulatory signals negatively inhibit T-cell function, called immune checkpoints.^3^ Peripheral tolerance operates by decreasing co-stimulatory signals and enhancing co-inhibitory signals. Negative regulatory lymphocytes, like Treg cells, also play an important role in the construction of peripheral tolerance.^4^

Cytotoxic T lymphocyte-associated antigen 4 (CTLA-4), programmed death 1 (PD-1) and programmed cell death ligand 1 (PD-L1) are the three most widely studied immune checkpoint molecules. CTLA-4 is expressed on the surface of activated T cells and is a critical regulator of T cells. It is homologous to CD28 and competitively binds to B7 ligands (CD80 or CD86). CTLA-4 also delivers inhibitory signals to T cells and hinders T cell activation. The expression level of CTLA-4 is transiently upregulated during T cell activation. CTLA-4 also has a higher affinity for B7 ligand than CD28, thus exerting a strong immunosuppressive effect.^5^ Furthermore, CTLA-4 is constitutively expressed on Treg cells, which mediate its immunosuppressive effects in peripheral tissues.^6^ PD-1 is a type I transmembrane protein that is mainly expressed on the surface of activated T cells, B cells, natural killer (NK) cells and other immune cells. Its ligands belong to the B7 family, including PD-L1 (B7-H1) and PD-L2 (B7-DC). PD-L1 is widely expressed on APCs, macrophages, endothelial cells, and various tissue cells.^7^ Tumor cells abnormally overexpress PD-L1, which binds to PD-1, leading to immune escape. Activation of the PD-1 signaling pathway mediates the phosphorylation of immunoreceptor tyrosine-based switch motif (ITSM), which in turn recruits Src homology-2 domain-containing protein tyrosine phosphatase-2 (SHP-2) protein and interrupts downstream signaling pathways, including the phosphoinositide 3-kinase (PI3K)/protein kinase B (AKT) pathway and RAS/mitogen-activated protein kinase kinase (MEK)/extracellular-signal regulated kinase (ERK) pathway. Abnormalities in these signaling pathways cause disorders of T-cell activation, proliferation, metabolism, and differentiation.^8^ Thus, the CTLA-4 pathway participates in the regulation of T cell maturation in lymphoid organs, while the PD-1 pathway is involved in the maintenance of peripheral tolerance.^9^

Immune checkpoint inhibitors (ICIs) are a class of monoclonal antibodies that target immune checkpoints and activate lymphocytes against tumors.^10^ ICIs are playing an increasingly important role in the field of cancer therapy and have brought considerable survival benefits to patients with advanced cancer. The most widely used ICIs are PD-1/PD-L1 inhibitors and CTLA-4 inhibitors.^11^ These ICIs have been approved for treating multiple tumors including melanoma, non-small cell lung cancer, and head and neck squamous carcinoma.^12^

## Pathogenesis of irAEs

**Fig. 1:**
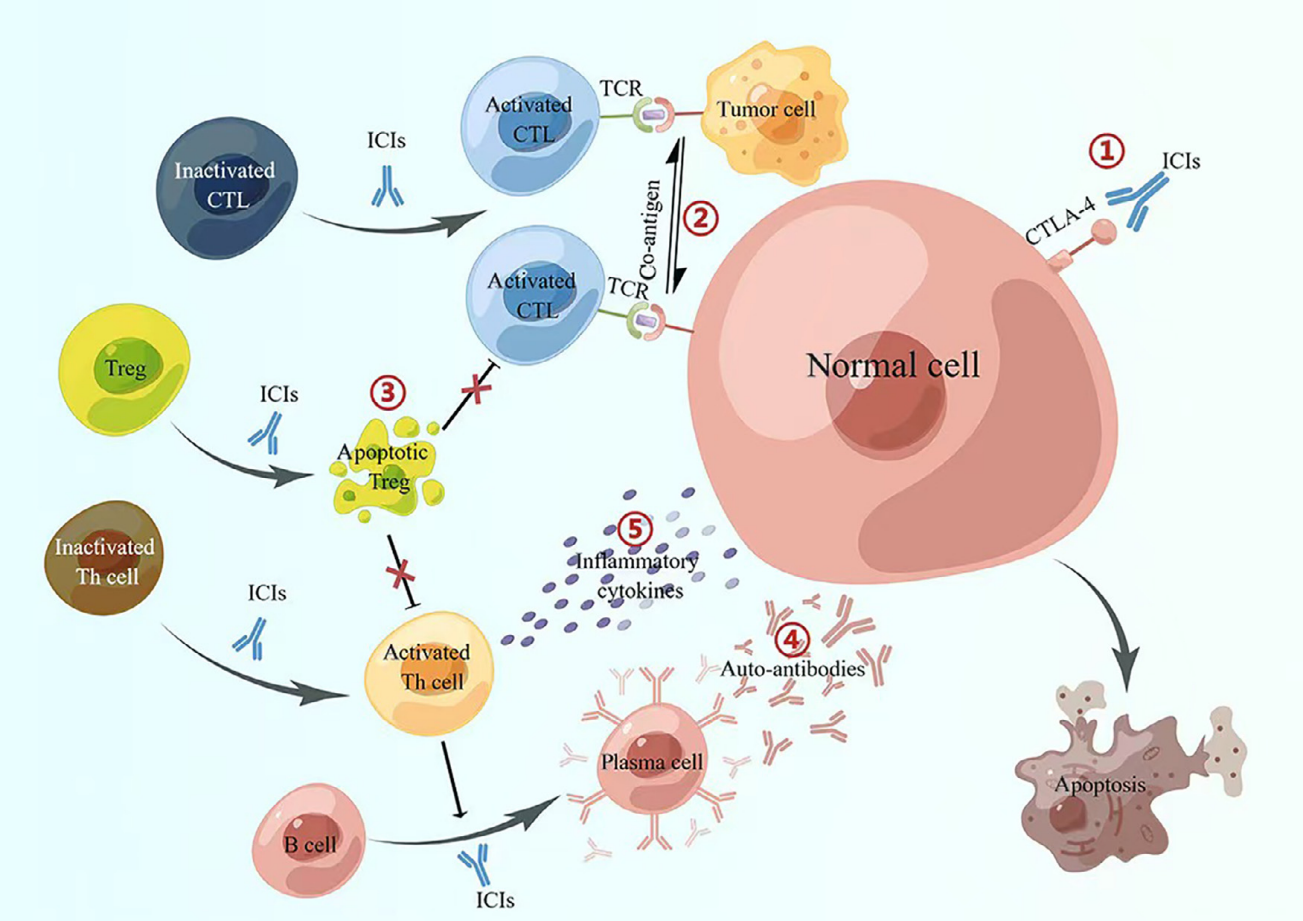
Major mechanisms of irAEs. (1) Off-targeting of ICIs; (2) co-antigens between self-antigens and tumor antigens; (3) dysfunction of immune negative regulatory cells; (4) B-cell activation and antibody-mediated injury; and (5) inflammatory cytokine-mediated injury. By Figdraw (www.figdraw.com). CTL: cytotoxic T lymphocytes; CTLA-4: Cytotoxic T lymphocyte-associated antigen 4; ICIs: Immune checkpoint inhibitors; irAEs: Immune-related adverse events; TCR: T-cell receptor; Th: T helper cell.

In addition to therapeutic effects against cancer, ICIs can also cause damage to normal tissues because of an induction of an imbalance to immune tolerance. These immune-related adverse events (irAEs) involve multiple organ systems throughout the body, including skin, gastrointestinal tract, liver, thyroid, lung, nerves, pancreas, kidney, and heart.^13^ The types of irAEs mediated by PD-1/PD-L1 inhibitors are slightly different from those of CTLA-4 inhibitors. Colitis, pituitary inflammation, and rash are more common in patients treated with CTLA-4 inhibitors, while pneumonia, arthritis, and abnormal thyroid function are more common with patients receiving PD-1/PD-L1 inhibitors.^14^ Currently, five mechanisms underlying irAEs have been described and are discussed in detail below [Fig. 1].

### Off-targeting of ICIs

ICIs may bind directly to normal tissues that express immune checkpoints and induce tissue damage. For example, pituitary endocrine cells are directly targeted by exogenous CTLA-4 inhibitors, which lead to pituitary parenchyma destruction and inflammation.^15^ Additionally, PD-L1 is expressed on a variety of tissues, such as oligodendrocytes, microglia, and islet cells.^16^ It is theoretically possible that PD-L1 inhibitors can directly bind these tissue cells.

### Co-antigens exist between self-antigens and tumor antigens

Neo-antigens generated by tumor mutations may share high homology with self-antigens expressed in normal tissues. Over-activated cytotoxic T lymphocytes (CTL) may recognize these normal tissues expressing co-antigens. A prospective cohort study of patients treated with PD-1 inhibitors highlighted the concept of co-antigens; in this study, 34.2% patients developed skin toxicity. Researchers performed matched analysis and found identical TCR sequences from tumor and lesioned skin.^17^ Another study of patients with ICI-mediated myocarditis similarly showed that tumor-infiltrating lymphocytes had similar selective clonal T-cell populations to myocardial-infiltrating lymphocytes.^18^ The application of ICIs may also induce epitope spreading, which is the process of immune response changes from initial targeted epitope-specific antigens to unselected self-antigens. During cancer treatment, the lysis of tumor cells leads to the release of massive proteins and antigens. These tumor antigens may be taken up by APCs and recruit more TCR-diverse T cells.^19^

### Dysfunction of immune negative regulatory cells

Studies have shown that Treg cells play a crucial role in maintaining immune homeostasis. These cells regulate the immune response by secreting anti-inflammatory cytokines (e.g., interleukin [IL]-10 and IL-35).^20^ Patients deficient in the Foxp3 gene (a marker of Treg cells) develop autoimmune disease.^21^ CTLA-4 is constitutively expressed on the surface of Treg cells, and CTLA-4 monoclonal antibody can induce the depletion of Treg cells through antibody-dependent cell-mediated cytotoxicity. Depletion of Treg cells causes an imbalance of peripheral immune tolerance and mediates the generation of irAEs.^22^

### B-cell activation and antibody-mediated injury

B cells also play an important role in the pathogenesis of irAEs. Studies have shown that ICI treatment leads to a series of changes in B cells, including a decrease in circulating B cells, and increase in CD21^low^ subpopulations and plasma cells.^23^ Additionally, the presence of autoantibodies was detected in many patients with irAEs such as thyroiditis, myasthenia gravis, and diabetes.^24^ A clinical study showed that patients with autoantibodies had a higher chance to experience irAEs.^25^ Therefore, the presence of autoimmune diseases is one of the contraindications for ICI treatment. PD-1 is expressed on the surface of various immune cells, whereas CTLA-4 is expressed only on the surface of activated cytotoxic T lymphocytes. Theoretically, CTLA-4 inhibitors do not directly target B cells and only affect them through T cells. In line with this, researchers found that irAEs mediated by autoantibodies were more common in patients treated with PD-1 inhibitors.^14^

### Inflammatory cytokine-mediated injury

The release of inflammatory cytokines may be the result of the immune response and an independent pathogenic factor that mediates tissue damage. Studies showed that irAEs usually occur with elevated inflammatory cytokines, such as IL-1, IL-6, IL-12, IL-23, IL-17, and tumor necrosis factor-α (TNF-α).^26^ Low baseline and progressively elevated cytokines after ICI treatment were also associated with the development of irAEs.^27^ The cytokines not only directly induce inflammatory responses and tissue damage, but also activate downstream signaling pathways.^28^ For example, IL-6 activates the Janus kinase (JAK)–signal transducer and activator of transcription (STAT) pathway in intrinsic immune cells to promote the gene transcription of factors with proliferative and inflammatory function.^29^^,^^30^ The Bruton's tyrosine kinase (BTK) pathway, which is regulated by IL-1, IL-6, and IL-12, plays an important role in the maturation, differentiation, and proliferation of B cells.^31^ The mitogen-activated protein (MAP) kinase-interacting kinase 1/2 (MNK1/2)–eukaryotic initiation factor 4E (eIF4E) pathway, which is associated with tumor cell proliferation and metastasis, is also regulated by cytokines.^32^ Phosphorylated eIF4E is a convergence point for many pro-tumor pathways as well as immunoregulatory pathways. Therefore, targeting the MNK1/2–eIF4E pathway may help inhibit tumor progression and control irAE-related inflammation.^33^

## Pathological characteristics of irAEs in different organ systems

Various irAEs are broadly mediated by the five mechanisms described above. However, irAEs from different organ systems hold their own pathological characteristics (Table 1).

**Table 1:** Representative mechanisms of different irAEs.

irAEs	Mechanisms	Representative characteristics
Skin toxicity		
Rash	Inflammatory cytokines	Infiltration of inflammatory cells (e.g., eosinophils)
Vitiligo	Cross-antigens	Common antigens between melanoma cells and melanocytes
Blister	Self-antibodies	
Hepatotoxicity	T-cell hyperactivation	Infiltration of immune cell (predominantly lymphocytes)
Gastrointestinal toxicity	Inflammatory cytokines	The levels of inflammatory cytokines such as IL-6, IL-17, and TNF-α were increased
Pulmonary toxicity	Inflammatory cytokines	The levels of C-reactive protein, IL-6, and anti-CD74 antibodies were increased
Endocrine toxicity		
Hypothyroidism	Self-antibodies	TPOAb and TgAb are elevated in many patients with ICIs-related hypothyroidism
Pituitaryitis	Off-targeting	CTLA-4 inhibitors bind directly to CTLA-4 in pituitary cells
Rheumatic toxicity	Self-antibodies	Antibodies anti-CCP or -RF
Neurotoxicity	Self-antibodies	Brain-reactive autoantibodies (anti-GABABR, -NMDAR, -MOG, -GFAP, -Zic4)
Cardiotoxicity	Cross-antigens	High overlap between the clonotypes of T cells in heart and tumor-infiltrating lymphocytes

CCP: Cyclic citrullinated peptide; CTLA-4: Cytotoxic T lymphocyte-associated antigen 4; GABABR: Type B gamma-aminobutyric acid receptor; GFAP: Glial fibrillary acidic protein; IL: Interleukin; irAEs: Immune-related adverse events; MOG: Myelin oligodendrocyte glycoprotein; NMDAR: N-methyl-D-aspartate receptor; RF: Rheumatoid factor; TNF-α: Tumor necrosis factor-α; TPOAb: Thyroid peroxidase antibody; TgAb: Thyroglobulin autoantibody.

### Skin toxicity

Skin toxicity is the most common irAE and can be broadly classified into four types on the basis of pathological histological patterns: inflammatory type, keratosis pilaris type, herpetic type, and melanocyte-associated type.^34^ The inflammatory type is characterized by maculopapular rash, with pathology showing epidermal spongiosis, papillary dermal edema, and perivascular lymphocytic and eosinophilic infiltration.^35^ The keratosis pilaris type is dominated by lichenoid rash or psoriasis. The pathology is characterized by hyperkeratosis, hyper-granulation, and serrated ridges.^36^ The herpetic type is a serious group of skin irAEs, mainly including bullous pemphigoid (BP), Stevens–Johnson syndrome, and drug-induced bullosa epidermolysis (TEN). BP is a subepidermal blistering disease mediated by autoantibodies against hemibridged granule antigens. Immunofluorescence shows a characteristic linear deposition of IgG and C3 at the top of the blister at the dermal–epidermal junction.^37^ Notably, nearly all cases of BP have been reported in patients treated with PD-1 or PD-L1 inhibitors, which may be related to the fact that CTLA-4 is only expressed on activated T cells. The melanocyte-associated type is represented by vitiligo, which occurs mainly in melanoma patients. The co-antigens between melanoma and melanocytes promote the development of vitiligo. The pathology exhibits dermal T cell infiltration with the absence of melanocytes.^38^ Studies have shown that pruritus is associated with eosinophil infiltration and elevated cytokines like IL-4. These patients may benefit from omalizumab (anti-immunoglobulin E [IgE] monoclonal antibodies) or IL-4 monoclonal antibodies.^39^

### Hepatotoxicity

Immune-mediated hepatotoxicity (IMH) presents as elevated transaminases with or without elevated bilirubin. Its histopathology can be broadly classified into two types: hepatocyte-dominated injury and bile duct-dominated injury. Hepatocellular injury is characterized by mixed lymphocyte-dominated immune cell infiltration throughout the lobules,^40^ while bile duct injury manifests as bile duct proliferation with mixed portal inflammation.^41^ Preclinical studies have shown the presence of CD8^+^ and CD4^+^ T cell infiltration in areas of hepatic necrosis. Further transcriptomic analysis indicated that apoptotic and necrotic pathways of hepatocytes were activated by inflammatory cytokines.^42^ Additionally, the infiltrating cells were predominantly CD8^+^ CTLs in CTLA-4 inhibitor-induced IMH, whereas CD8^+^ and CD4^+^ T lymphocytes were often co-existing in IMH mediated by PD-1/PD-L1 inhibitors. Scattered CD20^+^ B cells were also detected in some patients treated with PD-1 inhibitors.^43^

### Gastrointestinal toxicity

Immune-mediated colitis (IMC) is a serious irAE, which resembles inflammatory bowel disease in appearance under endoscopy. The main manifestations of IMC are mucosal edema, erythema, erosion, ulceration, and intestinal bleeding.^44^ The pathology is characterized by inflammatory cell infiltration in the lamina propria, dominated by lymphocytes, neutrophils, eosinophils, and plasma cells. Neutrophilic infiltration in the intraepithelial compartment as well as neutrophilic crypt abscesses is also common.^45^ Shahabi *et al*^46^ showed that increased expression of CD177 and carcinoembryonic antigen-related cell adhesion molecule 1 (CEACAM1) (neutrophil activation markers) was strongly associated with the development of IMC. Luoma *et al*^47^ showed an increased number of CTL and Ki-67^+^ T cells in IMC patients. Intestinal epithelial cells produce chemokines like C-X-C motif chemokine ligand 8 (CXCL8) and granulocyte macrophage-colony stimulating factor (GM-CSF) via the T helper cell 17 (Th17)/IL-17 pathway, which in turn induce neutrophil recruitment and infiltration. The infiltrating lymphocytes and neutrophils together mediate the inflammatory state of the intestine. Additionally, levels of inflammatory cytokines like IL-6, IL-17, and TNF-α were significantly increased in IMC patients. In addition to their own inflammation-inducing effects, these cytokines are able to amplify inflammation through a downstream cascade response. For example, IL-6 promotes the differentiation of naive CD4^+^ cells toward Th17 cells rather than Treg cells, which exacerbates the inflammatory injury.^48^ Overall, inflammatory cytokines play an important role in IMC. Monoclonal antibodies targeting cytokines (e.g., infliximab) may offer significant benefits for glucocorticoid-resistant IMC patients.

### Pulmonary toxicity

Checkpoint inhibitor-related pneumonitis (CIP) often occurs in lung cancer patients, with imaging chest CT showing interstitial lesions. Puncture biopsies are rarely performed in CIP patients, and therefore, the exploration of its pathogenesis relies on bronchoalveolar lavage (BAL).^49^ The prominent feature of BAL in CIP patients is lymphocytosis, but with limited predictive value. CTLA-4^+^ Treg cells have a potent negative regulatory effect on the inflammatory response mediated by CD8^+^ T cells and macrophages. Studies have shown that the increased lymphocytes in BAL from CIP patients mainly consist of CD4^+^ T cells. However, CTLA-4 and PD-1 expressions are significantly reduced in Treg cells.^50^ Researchers sequenced TCRs from lung-infiltrating T cells of CIP patients and found an overlap with tumor-infiltrating T cells. However, no overlap was found in peripheral blood T cells or secondary lymphoid organs.^51^ This indicated that both CTL-mediated cross-antigen reactions and Treg cells were involved in the development of CIP. Additionally, cytokine-induced injury plays an important role in CIP, in whom elevated C-reactive protein, IL-6, and anti-CD74 antibodies were detected.^52^ Tocilizumab validated that IL-6 plays an important role in the development of CIP, reflected in the good efficacy in its treatment of CIP.^53^ Because of the lack of biopsy tissue, no evidence of CIP directly mediated by off-targeting has been found.

### Endocrine toxicity

Immune-related endocrine toxicity mainly consists of pituitary inflammation, thyroid dysfunction, and insulin-deficient diabetes.^54^ Off-target effects and autoimmune antibodies may be the main pathogenesis of pituitary inflammation. Iwama *et al*^15^ found CD45^+^ monocyte infiltration in CTLA-4 inhibitor-mediated pituitary inflammation. CTLA-4 inhibitors bind to pituitary cells and directly damage them through antibody-dependent cell-mediated cytotoxicity. Additionally, autoimmune antibodies were detected in patients treated with CTLA-4 inhibitors. Thyroid dysfunction is more closely associated with autoimmune antibody-mediated damage. Thyroid peroxidase antibody (TPOAb) and thyroglobulin autoantibody (TgAb) are elevated in many patients with ICI-related hypothyroidism.^55^ However, not all patients showed auto-antibodies, suggesting that other modalities are involved in thyroid destruction. Insulin-deficient diabetes caused by ICIs is a rare but potentially life-threatening irAE. Pancreatic cell injury may be associated with autoimmune antibody. Approximately 50% of patients exhibited positive DM-related antibodies.^56^ Furthermore, almost all patients with immune-related diabetes were treated with PD-1 or PD-L1 inhibitors, which further indicated that the antibody-dependent pathway was the primary pathogenesis.

### Rheumatic toxicity

Immune-related rheumatologic toxicity is a group of irAEs that correlate with preexisting autoimmune diseases and mainly include inflammatory arthritis, myositis, vasculitis, and giant cell arteritis. The main manifestations are joint effusion and synovial thickening. Articular synovial fluid examination often shows inflammatory cell infiltration with a predominance of neutrophils.^57^ Similar to rheumatoid arthritis, autoimmune antibodies play an important role in ICI-mediated arthritis. Anti-cyclic citrullinated peptide (CCP) or -rheumatoid factor (RF) antibodies were found in some of these patients. However, the majority exhibited antibody negativity, suggesting the co-existence of other mechanisms. ICI-mediated myositis is mainly characterized by rapidly progressive proximal muscle weakness and elevated muscle enzymes.^58^ The pathological features showed multifocal necrosis of muscle fibers and inflammatory cell infiltration with a predominance of macrophages and lymphocytes.^18^ Notably, immune myositis tends to overlap with myocarditis or myasthenia gravis, rather than being independent.^59^ Therefore, special attention should be paid when relevant biomarkers are elevated. Immune-associated vasculitis is rare, with no significant differences in the clinical and pathological features compared with idiopathic vasculitis. The pathology of giant cell arteritis shows inflammatory cell infiltration in the arterial epithelium and muscular layer, along with arterial lumen narrowing.^60^

### Other toxicities

Other rare toxicities include cardiotoxicity, neurotoxicity, and nephrotoxicity. Myocarditis is a serious toxicity associated with ICIs that is usually fulminant and fatal.^61^ Patients mainly present with elevated cardiac enzymes and arrhythmias, including atrial fibrillation, ventricular arrhythmias, and conduction abnormalities.^62^ In a study involving 11 patients with myocarditis, the investigators found abundant infiltration of immune cells, mainly T cells and macrophages, but virtually no B cells.^18^ Deep sequencing of the complementarity-determining region 3 (CDR3) region of the TCR β chain revealed a high overlap between T cells in the heart and tumor-infiltrating T cells.^8^ This suggested that T cell-mediated cross-antigen responses play a crucial role in myocarditis. Neurotoxicity is closely related to antibody-mediated damage. The presence of autoantibodies, like anti-Hu antibodies and anti-striatal antibodies, has been reported.^63^ The pathogenesis of immune-associated nephrotoxicity, in which activated lymphocytes target self-antigens, was considered to be similar to that of autoimmune diseases. However, the pathology was not distinguishable from other drug-induced nephrotoxicity, which manifested as infiltration of T cells, eosinophils, and plasma cells in the renal interstitium.^64^

## Biomarkers for predicting irAEs

Currently, the most widely studied biomarkers for predicting irAEs are peripheral blood cells and cytokines. Other candidates include genomics of tumors, autoantibodies, and fecal microbiology. However, no biomarker has been shown to be an adequate predictor for irAEs to date.

In peripheral blood cells, high levels of lymphocytes and eosinophils and low levels of neutrophils/lymphocytes (NLR) were associated with the development of irAEs.^65^ Considering that the therapeutic effects of ICIs depend on lymphocytes, researchers have further explored the predictive role of different lymphocyte subsets. High CD4^+^T cell and low Treg cell levels were associated with immune-associated colitis, whereas high CD8^+^ CD38^+^ T cell levels were related to severe irAEs.^66^ Cytokines are the key regulators of the immune system and involved in multiple inflammatory responses. The predictive efficacy for irAEs is mainly reflected in its significantly elevated level after the application of ICIs. Currently, the cytokines with the highest correlation with irAEs include TNF-α, IL-6, and IL-17.^67^ Inhibitors targeting TNF-α and IL-6 have been clinically applied in the treatment of irAEs. Other candidate cytokine biomarkers being explored include IL-1, IL-2, IL-8, IL-12, IL-23, GM-CSF, interferon-γ (IFN-γ), C-X-C motif chemokine ligand (CXCL) 9, CXCL10, CXCL11, and C-C motif chemokine ligand 19 (CCL19).^68^

Tumor tissue-based biomarkers are currently used for the prediction of ICI efficacy. Studies have shown that there is a positive correlation between the efficacy of ICIs and irAEs. Patients who experienced irAEs were more likely to achieve a durable therapeutic effect. PD-L1 expression, tumor mutational burden (TMB), and microsatellite instability (MSI) are the three biomarkers used clinically to predict the efficacy of ICIs, and these also have a predictive role for the development of irAEs. Several studies have also shown the predictive efficacy of different combinations of genes, such as *LCP1* with *ADPGK* genes, for irAEs.^69^ Autoantibodies tend to be associated with organ-specific irAEs, such as anti-thyroid antibodies for thyroid irAEs, anti-bullous pemphigoid (BP) 180 immunoglobulin G (IgG) for skin irAEs, anti-G protein subunit alpha L (GNAL) antibodies for pituitary irAEs, and anti-CD74 for lung irAEs.^70^, ^71^, ^72^ A growing number of studies have demonstrated the relevance of fecal microbiology to irAEs, particularly for colitis. Increased *Enterococcus faecalis* and other thick-walled phyla were associated with the increased incidence of IMC, whereas increased anaphylactic phylum was related to the decreased incidence of IMC.^73^^,^^74^

## Therapeutic agents for irAEs and their mechanism

In general, patients with mild irAEs should undergo frequent organ function monitoring, while patients with severe irAEs should discontinue ICIs. Different therapeutic agents are available for different pathogenesis. The agents can be classified on the basis of their mechanisms, including non-specific immunosuppression, targeting T cells, targeting B cells and clearing autoimmune antibodies, and targeting inflammatory cytokines and their downstream signaling pathways (Table 2).

**Table 2:** Therapeutic agents for irAEs.

Agent	Application status	Recommended level	Mechanism
Non-specific immunosuppressive			
Glucocorticoids	All kinds of irAEs	★★★★★	Broad-spectrum anti-inflammatory and non-specific immunosuppression
MMF	Glucocorticoid resistance	★★★★	Removal of guanine nucleotides from T cells and B cells to inhibit cell proliferation
Targeting T cells			
IVIGs	Glucocorticoid resistance	★★★★	Inhibit T/B cell activation, neutralize autoantibodies, and regulate cytokines
Tacrolimus	Hepatotoxicity	★★★	Calcium-regulated phosphatase inhibitor, inhibits T-cell activation
ATG	Hepatotoxicity, hematotoxicity, cardiotoxicity	★★★	Induction of T-cell depletion by complement-dependent cytolysis
Abatacept	Cardiotoxicity	★★	Soluble CTLA-4 fusion protein that can induce T cell dysfunction
Vedolizumab	Gastrointestinal toxicity	★★★★	Block integrin α4β7 expressed in the intestinal epithelium to reduce T-cell infiltration
Natalizumab	Preclinical	★	Block integrin α4 expressed in the blood–brain barrier to reduce T-cell infiltration
Targeting B cells and antibodies			
Rituximab	Hematotoxicity, neurotoxicity	★★★	Targeting CD20 to induce the depletion of B cells
Plasma replacement	Rheumatic toxicity, neurotoxicity	★★★	Remove pathogenic autoantibodies
Targeting cytokines			
Infliximab	Gastrointestinal and pulmonary toxicity	★★★★	Targeting TNF-α
Adalimumab	Alternatives to infliximab	★★	
Golimumab	Alternatives to infliximab	★★	
Certolizumab	Alternatives to infliximab	★★	
Etanercept	Alternatives to infliximab	★★	
Tocilizumab	Pulmonary and rheumatic toxicity	★★★	Targeting IL-6R
Omalizumab	Skin toxicity	★★★	Targeting IgE, for skin toxicity
Anakinra	Preclinical	★	Targeting IL-1
Rilonacept	Preclinical	★	
Canakinumab	Preclinical	★	
Secukinumab	Preclinical	★	Targeting IL-17
Ustekinumab	Preclinical	★	Targeting IL-12/23
Risankizumab	Preclinical	★	
Targeting signaling pathways			
Tofacitinib	Cardiotoxicity	★★	Targeting JAK–STAT pathway

ATG: Anti-thymocyte globulin; CTLA-4: Cytotoxic T lymphocyte-associated antigen 4; IgE: Immunoglobulin E; IL: Interleukin; irAEs: Immune-related adverse events; IVIGs: Intravenous immunoglobulins; JAK: Janus kinase; MMF: Mycophenolate mofetil; STAT: Signal transducer and activator of transcription; TNF-α: Tumor necrosis factor-α.

### Non-specific immunosuppressive agents

Non-specific immunosuppression and anti-inflammation are the most important modalities for irAE treatment. Glucocorticoids, the most widely used agent for irAEs, inhibit the activation, proliferation, and differentiation of immune cells and modulate inflammatory cytokines.^75^ Glucocorticoids exerts anti-inflammatory effects through cytosolic glucocorticoid receptor α (cGCRα), which promotes the transcription and translation of anti-inflammatory proteins. Additionally, glucocorticoids inhibit the production of adhesion molecules and reduce vascular permeability, leading to a decrease in inflammatory exudation.^76^ The initial dose for glucocorticoids is usually 1–2 mg/kg for grade 3–4 irAEs, except for life-threatening irAEs like severe myocarditis. When irAEs cannot be effectively controlled with glucocorticoids alone, the addition of other non-specific immunosuppressive agents such as mycophenolate mofetil (MMF) should be considered. MMF can be rapidly hydrolyzed into its active metabolite (mycophenolic acid) after entry into the body, which selectively inhibits the proliferative function of lymphocytes by removing guanine nucleotides.^77^ Non-specific immunosuppression agents also include intravenous immunoglobulins (IVIG), which contain a variety of IgG antibodies that can inhibit B cell activation and antibody production, neutralize pathogenic autoantibodies, induce auto-reactive T cell inactivation, regulate cytokines, interfere with complement activation, and inhibit macrophage and dendritic cell function.^78^

### Agents targeting T cells

Among all immune cells, T cells play the most crucial role in the development of irAEs. Specifically targeting T cells, including inhibiting T cell function, promoting T cell depletion, and reducing T cell infiltration, can effectively reduce irAEs. Tacrolimus, a calcium-regulated phosphatase inhibitor, suppresses T cell activation by inhibiting calcium-dependent signaling pathways.^79^ Anti-thymocyte globulin (ATG) is a cytotoxic antibody that exerts its immunosuppressive effects by destroying lymphocytes in the recirculating pool through antigen-dependent cell-mediated cytotoxicity.^80^ However, ATG is not used as a routine therapeutic strategy for irAEs because of its strong immunosuppressive effect, which may cause rapid tumor progression and increase the risk of infection. Abatacept is a soluble CTLA-4 fusion protein that induces T-cell deactivation but carries the same risk of impairing antitumor efficacy. Additionally, the local inflammatory response can be alleviated by reducing the infiltration of T cells from the blood circulation to peripheral tissues. Vedolizumab blocks integrin α4β7, which is expressed in the intestinal epithelium, while natalizumab blocks integrin α4 on the blood–brain barrier. Inhibition of integrin function can reduce T-cell infiltration in the intestine and cranium. These antibodies have been successfully applied in the treatment of corticosteroid-refractory immune colitis and encephalitis.^81^

### Agents targeting B cells and antibodies

In addition to T cells, B cells also play an important role in irAEs. Targeting B cells and clearing autoimmune antibodies can help control irAEs.^82^ Rituximab is a monoclonal antibody that targets CD20, a biomarker of B cells, and induces the depletion of B cells. Rituximab has been recommended for the treatment of autoimmune antibody-positive immune-associated encephalitis.^83^ Additionally, preclinical studies showed that B-cell depletion did not impede the antitumor efficacy of PD-1 inhibitors.^84^ Plasma replacement can assist in clearing pathogenic autoantibodies and has been used to treat serious irAEs.^85^

### Agents targeting cytokines

In recent years, there has been an increasing focus on personalized irAE management targeting inflammatory cytokines. Studies have shown that irAEs often occur with elevated levels of cytokines like TNF-α and IL-6. TNF-α is mainly secreted by activated macrophages and elevated in the acute phase of the inflammatory response. It can mediate a reduction in lysosomal stability and cause cytolysis, thus promoting the killing function of immune cells against tumors. TNF-α also damages endothelial cells and leads to vascular dysfunction, promotes neutrophil adhesion, and stimulates the local inflammatory response.^86^ Infliximab is a human-mouse chimeric TNF-α monoclonal antibody that binds to TNF-α with high affinity. Infliximab has been approved for treating severe autoimmune diseases such as rheumatoid arthritis, Crohn's disease, and psoriasis.^87^ For irAEs, infliximab was recommended for the post-line therapy of rheumatic or gastrointestinal toxicity. The application of infliximab has also been reported in the treatment of other severe irAEs such as myocarditis, neurotoxicity, and nephrotoxicity.^88^ Other inhibitors of TNF-α include adalimumab, golimumab, certolizumab, and etanercept. Despite the absence of large-scale clinical evidence, these treatments may also be considered as comparable alternatives when infliximab is not available.

IL-6 is secreted by T cells, B cells, monocytes, and fibroblasts. It is involved in multiple physiological processes of inflammation, including T cell activation, immunoglobulin secretion, and hematopoietic precursor cell proliferation.^89^ Notably, IL-6 is not only involved in the regulation of inflammatory responses, but it also promotes tumor growth, invasion, and metastasis.^90^ Tocilizumab is a recombinant humanized anti-IL-6 receptor monoclonal antibody. In a retrospective study, tocilizumab improved irAEs symptoms in 79.4% of patients with no significant effect on survival prognosis.^91^ Therefore, tocilizumab has been recommended in the guidelines as one of the alternative therapeutic agents for steroid-refractory irAEs. Studies targeting other cytokines for treating irAEs are ongoing. These studies are exploring omalizumab (targeting IgE) for skin toxicity,^91^ anakinra (targeting IL-1) for rheumatologic toxicity,^[^^92^^]^ ustekinumab (targeting IL-12/23) for psoriatic arthritis,^93^ and secukinumab (targeting IL-17) for psoriasis.^94^

### Agents targeting signaling pathways

In addition to targeting inflammatory cytokines, the downstream regulated signaling pathways have also become a focus of interest. Studies have shown that the JAK–STAT pathway is closely associated with both adaptive and intrinsic immune responses.^95^ Inflammatory cytokines, like IL-6 and IL-17, bind to immune cells and activate the JAK–STAT signaling pathway. Phosphorylated STAT translocates to the nucleus, promoting inflammation-related gene transcription and translation.^96^ JAK inhibitors inhibit the JAK–STAT pathway and thus suppress the secretion of IL-1, IL-2, IL-6, CXCL9, and CXCL12. These agents may contribute to immune modulation and attenuate irAEs.^97^ Recently, cases of immune-associated colitis, myocarditis, and arthritis treated with JAK inhibitors (tofacitinib) with promising efficacy have been reported.^98^ However, blocking JAK–STAT pathway may affect the antitumor efficacy of ICIs.^99^ Whether the application of JAK–STAT inhibitors can be extended in the field of irAEs is unclear, and more clinical studies are needed to explore its potential benefits and risks. irAEs are also related to other signaling pathways, like the MNK pathway and BTK pathway.^28^ However, no blocking agents are currently available in clinical practice.

## Challenges and perspectives

At present, the pathogenesis of irAEs for various organ systems remains unclear. IrAEs related to different organ systems may involve separate mechanisms and thus require different therapeutic strategies. Five mechanisms of irAEs have been recognized, including off-targeting of ICIs, co-antigens between self-antigens and tumor antigens, dysfunction of immune negative regulatory cells, B-cell activation and antibody-mediated injury, and inflammatory cytokine-mediated injury. A positive correlation between the occurrence of irAEs and the efficacy of ICIs has been recognized. However, many patients have died from severe irAEs or tumor rapid progression resulting from long-term cessation of tumor therapy. Biomarkers for the prediction of irAEs include PD-L1, various types of peripheral blood cells, autoantibodies, cytokines, gut microflora, gene mutation profiles, and TMB.^68^ However, no biomarker that can predict the occurrence of irAEs with high accuracy has been identified. Non-specific immunosuppressive agents, represented by glucocorticoids, are the primary therapy for irAEs. However, this treatment inevitably causes tumor progression because of immunosuppression. Cytokine-based personalized therapeutic models are currently available for limited cytokines such as TNF-α and IL-6. Therefore, future research should focus on accurate prediction of the adverse effects before drug administration and personalized therapeutic strategies. Additionally, pathological biopsies can help to clarify the diagnosis of irAEs. However, the value for guiding individualized drug administration is still limited. Therefore, it is necessary to further explore the association between pathological typing and the pathogenesis of irAEs as well as personalized drug regimens.

## Conflicts of interest

None.
